# Bayesian clustering prior with overlapping indices for effective use of multisource external data

**DOI:** 10.1177/09622802251367439

**Published:** 2025-09-15

**Authors:** Xuetao Lu, J Jack Lee

**Affiliations:** Department of Biostatistics, The University of Texas MD Anderson Cancer Center, USA

**Keywords:** Information borrowing, real-world data, evidence synthesis, heterogeneity, clustering, prior data congruency, prior robustness

## Abstract

The use of external data in clinical trials offers numerous advantages, such as reducing enrollment, increasing study power, and shortening trial duration. In Bayesian inference, information in external data can be transferred into an informative prior for future borrowing (i.e. prior synthesis). However, multisource external data often exhibits heterogeneity, which can cause information distortion during the prior synthesizing. Clustering helps identifying the heterogeneity, enhancing the congruence between synthesized prior and external data. Obtaining optimal clustering is challenging due to the trade-off between congruence with external data and robustness to future data. We introduce two overlapping indices: the overlapping clustering index and the overlapping evidence index . Using these indices alongside a K-means algorithm, the optimal clustering result can be identified by balancing this trade-off and applied to construct a prior synthesis framework to effectively borrow information from multisource external data. By incorporating the (robust) meta-analytic predictive (MAP) prior within this framework, we develop (robust) Bayesian clustering MAP priors. Simulation studies and real-data analysis demonstrate their advantages over commonly used priors in the presence of heterogeneity. Since the Bayesian clustering priors are constructed without needing the data from prospective study, they can be applied to both study design and data analysis in clinical trials.

## 1. Introduction

Incorporating multisource external data into the design and analysis of clinical trials has become a field of significant interest. U.S. Food and Drug Administration (FDA) has issued the guidance, “Use of Real-World Evidence to Support Regulatory Decision-Making for Medical Devices,”^1^ to encourage the integration of external data in new studies. This practice aims to enhance the efficiency of clinical trials by leveraging real-world data (RWD) to inform study parameters, potentially reducing sample size, increasing the statistical power and precision of testing or estimating study outcomes, and accelerating trial timelines.^2^ In the Bayesian framework, a critical component of incorporating RWD is the construction of informative priors from external data, a process commonly referred to as evidence synthesis. Methods of synthesizing informative priors have been extensively studied. For instance, the meta-analytic predictive (MAP) prior^3,4^ uses meta-analysis to summarize information from external data into an informative prior. The power prior (PP)^5,6^ adjusts the influence of external data on the analysis of new data by applying a likelihood discounting approach based on the relevance and reliability of the external data. Commensurate priors^7,8^ and multisource exchangeability models (MEMs)^9^ modulate the degree of information borrowing from external data according to their relevance and congruence to the new data. The elastic prior^10^ dynamically borrows information from external data using a monotonic function of a congruence measure between external and new data.

The use of prior information is critical in both trial design and data analysis. During the trial design stage, where new trial data is not yet available, prior information is typically derived from domain knowledge or external data. In the data analysis stage, once new trial data is available, priors can be refined by evaluating the similarity between the external and new trial data. Constructing an informative prior that is suitable for both trial design and data analysis is challenging. For example, with the exception of the MAP prior, all of the aforementioned priors require the new trial data, which limits their applicability during the trial design stage.

Another challenge arises from the diversity and heterogeneity of multiple data sources, including differences in study design, population characteristics, eligibility criteria, outcome measures, etc.^4^ Such variability complicates the accurate transferring or borrowing of information from external data into a prior, potentially leading to information distortion and adversely affecting the analysis of the new trial. Therefore, it is crucial to accurately identify the heterogeneity across external datasets. MAP (or rMAP) prior accommodates this heterogeneity by using the linear mixed model with a random effect parameter.^11^ However, a single parameter may not adequately capture complex heterogeneity structures, such as scenarios where external datasets include multiple clusters with varying degrees of homogeneity. The MEM approach attempts to address this challenge by measuring pairwise exchangeability among the interested random parameters (associated with external datasets). However, it does not explicitly construct a prior that adapts to the heterogeneity structure. in this paper, we focus on clustering methods, where heterogeneity is identified by partitioning the interested random parameters (associated with external datasets) into distinct clusters. Bayesian nonparametric clustering with Dirichlet process^12^ is a widely used approach. For example, based on it, Chen and Lee^13^ proposed a Bayesian clustering hierarchical model to dynamically partition sub-trials into clusters for efficient information borrowing in basket trials. Nevertheless, in their model, the number of clusters is determined by a hyperparameter, making it challenging to establish an interpretable and unified criterion for selecting the optimal hyperparameter value across different applications.^14^ Additionally, based on our knowledge, this approach has not been applied to prior synthesis with multisource external data.

To address these challenges, we propose a novel approach for synthesizing informative priors from multisource external data, leveraging the concept of overlapping coefficients.^15,16^ Specifically, we introduce the overlapping clustering index (OCI) and employ a K-means algorithm to identify heterogeneity across external datasets. To measure the congruence between the synthesized prior and external data, we define an overlapping evidence index (OEI). A higher OEI indicates more accurate information transferred from external data to the prior, reflecting stronger congruence between them. There is, however, an inherent trade-off between maximizing OEI and ensuring the robustness of the prior to new data. To address this, we propose an OEI-based criterion that balances this trade-off, enabling accurate heterogeneity identification through optimal clustering. Compared to existing nonparametric clustering methods,^17,18^ our criterion is more interpretable and maintains a consistent standard across different applications. Using the optimal clustering results, we introduce the Bayesian clustering prior, a flexible framework for prior synthesis. By integrating the MAP and robust MAP priors within this framework, we develop the Bayesian clustering MAP (BCMAP) and robust Bayesian clustering MAP (rBCMAP) priors. They exhibit desirable properties and are applicable in both trial design and data analysis stages.

Section 2 introduces the notations, assumptions, and key challenges of Bayesian evidence synthesis with multisource external data. In Section 3, we propose two overlapping indices, explore the trade-off between evidence congruence and robustness, and present a K-means algorithm to achieve optimal clustering for accurate heterogeneity identification. Section 4 details the construction of Bayesian clustering priors, provides a sensitivity analysis and OEI-based threshold selection guidelines, and includes an illustrative example. Simulation studies comparing our method with existing approaches are presented in Section 5. Section 6 demonstrates the application of the proposed method to two real-world external datasets, featuring binary and continuous endpoints. Section 7 concludes with a brief discussion. To facilitate reading and referencing, Table 5 in the Appendix lists the symbols and abbreviations used in this paper.

## 2. Bayesian evidence synthesis from multisource external data

Let $Y_{1},\ldots ,Y_{H}$ denote external data from multiple sources. Assume θ to be the common parameter of interest. Bayesian evidence synthesis aims to create an informative prior of θ from $Y_{1},\ldots ,Y_{H}$ , denoted as $\pi (\theta |Y_{1},\ldots ,Y_{H})$ . Then, for any new data $Y^{*}$ , the inference of θ can borrow information from $\pi (\theta |Y_{1},\ldots ,Y_{H})$ through $p(\theta |Y^{*})\propto L(Y^{*}|\theta )\pi (\theta |Y_{1},\ldots ,Y_{H})$ , where $L(Y^{*}|\theta )$ is the likelihood function. In this paper, we assume $Y^{*}$ is unknown; in other words, $Y^{*}$ has no effect on the construction of $\pi (\theta |Y_{1},\ldots ,Y_{H})$ . In Bayesian inference, the prior distribution represents the beliefs or information about a parameter before observing the new data. Avoiding “use the data twice”^19^ is fundamental for maintaining the integrity of the Bayesian updating process. Compared to the most existing methods, such as the MEM and power prior, which use $Y^{*}$ first in the prior and then in the likelihood, our approach strictly follows the rule that do not use the data twice. It makes $\pi (\theta |Y_{1},\ldots ,Y_{H})$ applicable in both trial design (without $Y^{*}$ ) and data analysis (with $Y^{*}$ ). Another assumption in this paper is the exclusion of covariate information. This assumption stems from the practical challenges of obtaining such information. For instance, patient-level data may be restricted from the public access, even for research purposes. Even when this information is accessible, the available covariates often vary across data sources. For example, data source 1 includes covariates $X_{1},X_{2},X_{3}$ , data source 2 include $X_{2},X_{3},X_{5}$ , and data source 3 include $X_{3},X_{4},X_{5}$ , leaving only $X_{3}$ as a common covariate. In such cases, inference relying solely on $X_{3}$ may lead to questionable conclusions.

**Figure 1.**
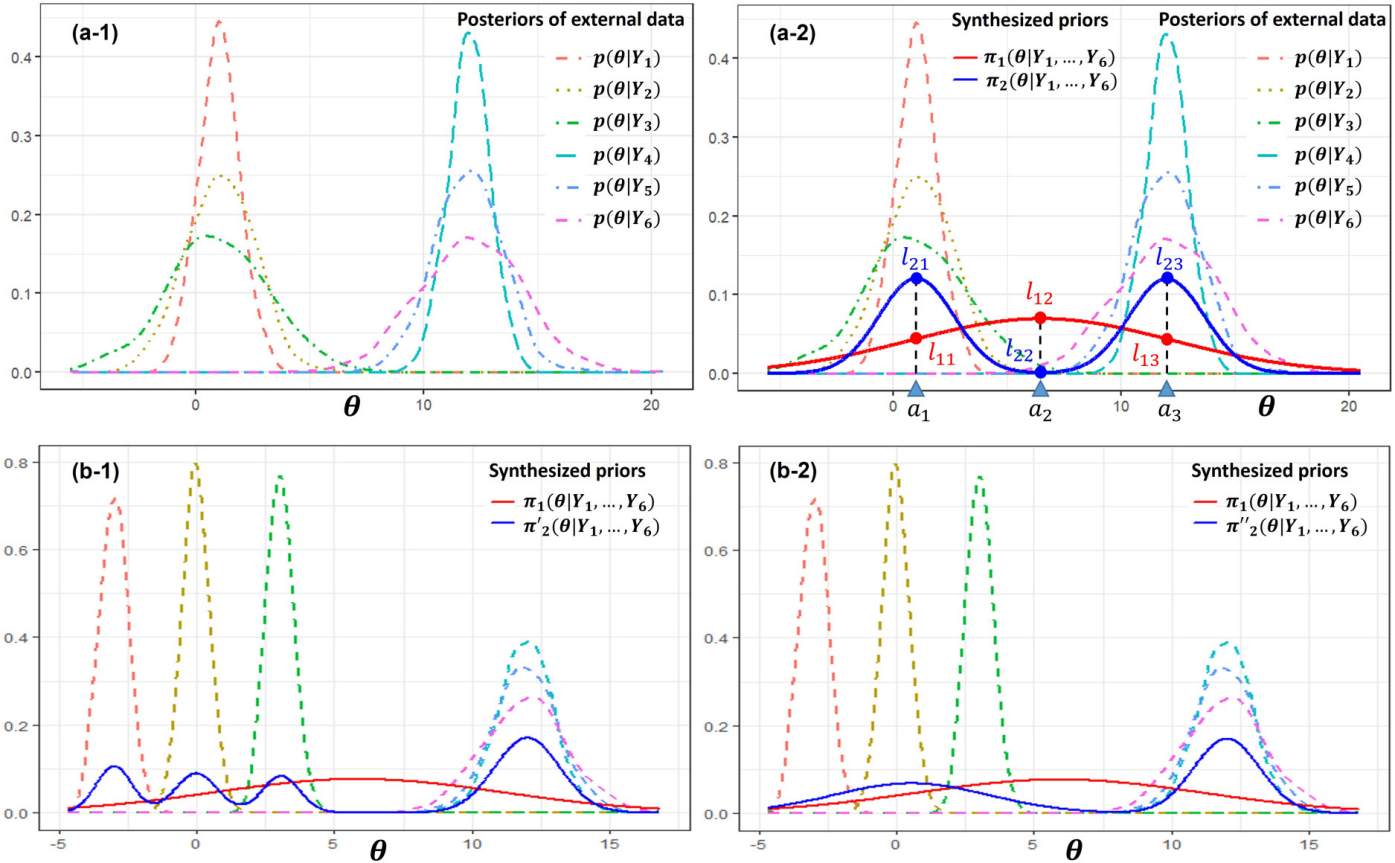
Information distortion and the trade-off between evidence congruence and robustness. Panel (a-1) shows an example with the posterior distributions of six external datasets. Panel (a-2) illustrates the distortion of information due to heterogeneity, demonstrating that prior $\pi_{2}$ , which correctly accounts for heterogeneity, is more appropriate than prior $\pi_{1}$ . Panels (b-1) and (b-2) demonstrate the trade-off between evidence congruence and robustness. In Panel (b-1), the prior $\pi_{2}^{\prime }$ has stronger evidence congruence but weaker robustness. In Panel (b-2), the prior $\pi_{2}^{\prime \prime }$ has weaker evidence congruence but stronger robustness.

In practice, heterogeneity often exists among data sources, such as multi-regional data or data from multiple health centers. One approach to address this issue is to filter out certain datasets to achieve homogeneity. However, without knowledge of covariate information or the new data $Y^{*}$ , this method risks losing valuable information, furthermore it is hard to determine which data sources should be excluded. An alternative approach is to include all external datasets. In this scenario, the evidence synthesis model used to construct the prior must be capable of effectively identifying the heterogeneity across the various data sources and be able to handle it well. Otherwise, it may distort the information transferred from $Y_{1},\ldots ,Y_{H}$ to $\pi (\theta |Y_{1},\ldots ,Y_{H})$ . The information of θ in $Y_{1},\ldots ,Y_{H}$ is contained in $p(\theta |Y_{1}),\ldots ,p(\theta |Y_{H})$ where $p(\theta |Y_{h})$ , h=1,…,H , is obtained through $p(\theta |Y_{h})\propto L(Y_{h}|\theta )\pi (\theta )$ , and π(θ) can be either a weakly informative or an informative prior of θ . (Note: Instead of using $\theta_{1},\ldots ,\theta_{H}$ , we utilize the posteriors $p(\theta |Y_{1}),\ldots ,p(\theta |Y_{H})$ to reflect the heterogeneity of the external data. This notation emphasizes the congruence between the external data and the synthesized prior $\pi (\theta |Y_{1},\ldots ,Y_{H})$ .) The information distortion can be illustrated by the example shown in Panels (a-1) and (a-2) of Figure 1. The example includes six external datasets with corresponding posteriors $p(\theta |Y_{1}),\ldots ,p(\theta |Y_{6})$ as shown in Panel (a-1). It is obvious that these datasets are heterogeneous and can be partitioned into two clusters. In Panel (a-2), we examine information transfer at three points: $\theta =a_{1}$ , $\theta =a_{2}$ , and $\theta =a_{3}$ . The transferred information is quantified using the likelihood under priors $\pi_{1}$ and $\pi_{2}$ . The corresponding likelihoods are $l_{11},l_{12},l_{13}$ for $\pi_{1}$ and $l_{21},l_{22},l_{23}$ for $\pi_{2}$ . According to the posteriors $p(\theta |Y_{1}),\ldots ,p(\theta |Y_{6})$ , it is clear that the likelihoods at $\theta =a_{1}$ and $\theta =a_{3}$ should be greater than the likelihood at $\theta =a_{2}$ . However, $\pi_{1}$ performs oppositely, with $l_{11},l_{13}<l_{12}$ . This occurs because the synthesizing method of $\pi_{1}$ fail to identify the heterogeneity, thus distorting the information in the external data. Conversely, $\pi_{2}$ correctly captures the heterogeneity and accurately reflects the information in the external data, with $l_{21},l_{23}>l_{22}$ .

As discussed above, the quality of a synthesized prior can be evaluated by the congruence of information about θ between $Y_{1},\ldots ,Y_{H}$ and $\pi (\theta |Y_{1},\ldots ,Y_{H})$ . We refer to this congruence as the evidence congruence of $\pi (\theta |Y_{1},\ldots ,Y_{H})$ with respect to $Y_{1},\ldots ,Y_{H}$ . In Panel (a-2) of Figure 1, it is clear that $\pi_{2}$ has stronger evidence congruence than $\pi_{1}$ . However, higher evidence congruence is not always better. Robustness is another crucial criterion for evaluating the quality of a synthesized prior.^4^ As shown in Panels (b-1) and (b-2), $\pi_{2}^{\prime }$ is constructed by identifying four clusters in the external datasets, whereas $\pi_{2}^{\prime \prime }$ assumes two clusters. It is easy to check that the evidence congruence of $\pi_{2}^{\prime }$ is greater than that of $\pi_{2}^{\prime \prime }$ . But we prefer $\pi_{2}^{\prime \prime }$ because it is more robust. In sum, both criteria of evidence congruence and robustness are closely related to the heterogeneity identification. Since $p(\theta |Y_{1}),\ldots ,p(\theta |Y_{H})$ contain all the information about θ , an accurate clustering of $p(\theta |Y_{1}),\ldots ,p(\theta |Y_{H})$ can strike a good balance between evidence congruence and robustness, thereby helping to create a high-quality informative prior.

## 3. Overlapping indices

Overlapping coefficient (OVL) is a measure of the intersection area between two probability density or mass functions. Let X and Y be two random variables with probability density or mass functions f and g , respectively. Ω is the common support of f and g . OVL can be defined by equation (1). (Note: the integral expression is used in this paper without loss of generality)

(1) $$\mathrm{OVL}(X,Y)=\left\{ \begin{array}{ll} \int_{\Omega }min\{f(t),g(t)\}\;dt, & \text{continuous}\\ \sum_{\Omega }min\{f(t_{i}),g(t_{i})\}\cdot I(t_{i}\in \Omega ), & \text{discrete} \end{array}\right.$$

Based on the concept of OVL, we propose two overlapping indices for the clustering of $p(\theta |Y_{1}),\ldots ,p(\theta |Y_{H})$ to address the challenges discussed in Section 2.

### 3.1. Overlapping clustering index and K-means clustering

Let $p(\theta |Y_{h})$ , h=1,…,H , be the probability density function (pdf) or probability mass function (pmf) of posterior distributions obtained from the external datasets $Y_{1},\ldots ,Y_{H}$ . The corresponding random variables are denoted as $\theta |Y_{h}$ , h=1,…,H . A partition S groups them into K clusters $\left\{G_{1},\ldots ,G_{K}\right\}$ , 1≤K≤H . For each cluster $G_{m}$ , m=1,…,K , let $\Theta_{m}$ be a Gaussian random variable with density function $g_{m}$ , which is the maximum likelihood estimation (MLEs of mean and variance) obtained from the samples of the random variables $\theta |Y_{h}$ in cluster $G_{m}$ . Then, the overlapping clustering index (OCI) of this partition K is defined as follows:

(2) $${\mathrm{OCI}}_{K}=\sum_{m=1}^{K}\sum_{h=1}^{H}\mathrm{OVL}(\theta |Y_{h},\Theta_{m})\cdot I(\theta |Y_{h}\in G_{m}),$$

where I(⋅) is a indicator function. The random variables $\theta |Y_{h}$ in the same cluster $G_{m}$ are assumed to be exchangeable. $\Theta_{m}$ denotes the centroid of cluster $G_{m}$ , and it is modeled as a Gaussian random variable. This assumption is justified by the central limit theorem, which implies that as the size of $G_{m}$ increases, the mean of the all posteriors within the cluster will approximate a Gaussian distribution, regardless of the differences among $\theta |Y_{h}$ , h=1,…,H .

${\mathrm{OCI}}_{K}$ measures the overall within cluster homogeneity of the K-partition. Based on it, we can define the optimal $S_{K}^{(\mathrm{oci})*}$ for K partition as follows:

(3) $$\begin{array}{rl} S_{K}^{(\mathrm{oci})*} & =\underset{S_{K}}{\arg\;\max}\;{\mathrm{OCI}}_{K}\\ & =\underset{S_{K}}{\arg\;\max}\;\sum_{m=1}^{K}\sum_{h=1}^{H}\mathrm{OVL}(\theta |Y_{h},\Theta_{m})\cdot I(\theta |Y_{h}\in G_{m}^{*}). \end{array}$$

With $S_{K}^{(\mathrm{oci})*}:\{G_{1}^{*},\ldots ,G_{K}^{*}\}$ , we can calculate the corresponding ${\mathrm{OCI}}_{K}^{*}$ :

(4) $${\mathrm{OCI}}_{K}^{*}=\sum_{m=1}^{K}\sum_{h=1}^{H}\mathrm{OVL}(\theta |Y_{h},\Theta_{m}^{*})\cdot I(\theta |Y_{h}\in G_{m}^{*}).$$

An optimal clustering $S_{K}^{(\mathrm{km})*}$ can be found through the following K-means algorithm:

(5) $$S_{K}^{(\mathrm{km})*}=\underset{S_{K}}{\arg\;\min}\;\sum_{m=1}^{K}\sum_{h=1}^{H}d(\theta |Y_{h},\Theta_{m})\cdot I(\theta |Y_{h}\in G_{m}^{*})$$

where $d(\theta |Y_{h},\Theta_{m})=1-\mathrm{OVL}(\theta |Y_{h},\Theta_{m})$ is a distance measure between two random variables $\theta |Y_{h}$ and $\Theta_{m}$ . The equivalence between $S_{K}^{(\mathrm{oci})*}$ and $S_{K}^{(\mathrm{km})*}$ is shown as follows:

$$\begin{array}{rl} S_{K}^{(\mathrm{oci})*} & =\underset{S_{K}}{\arg\;\min}\;\sum_{m=1}^{K}\sum_{h=1}^{H}d(\theta |Y_{h},\Theta_{m})\cdot I(\theta |Y_{h}\in G_{m}^{*})\\ & =\underset{S_{K}}{\arg\;\min}\;\sum_{m=1}^{K}\sum_{h=1}^{H}[1-\mathrm{OVL}(\theta |Y_{h},\Theta_{m})]\cdot I(\theta |Y_{h}\in G_{m}^{*})\\ & =\underset{S_{K}}{\arg\;\max}\;\sum_{m=1}^{K}\sum_{h=1}^{H}\mathrm{OVL}(\theta |Y_{h},\Theta_{m})\cdot I(\theta |Y_{h}\in G_{m}^{*})=S_{K}^{(\mathrm{km})*}. \end{array}$$

The above mentioned K-means algorithm closely resembles the standard K-means algorithm. The differences lie in the definitions of the centroid $\Theta_{m}$ and the distance $d(\theta |Y_{h},\Theta_{m})=1-\mathrm{OVL}(\theta |Y_{h},\Theta_{m})$ . A detailed description of the proposed K-means algorithm in the form of pseudo-code can be found in Algorithm 1 in the Appendix.

### 3.2. Overlapping evidence index and trade-off between evidence congruence and robustness

For any fixed K , the heterogeneity among $p(\theta |Y_{1})$ ,…, $p(\theta |Y_{H})$ can be effectively identified through $S_{K}^{(\mathrm{oci})*}$ . As demonstrated by Figure 1 in Section 2, the evidence congruence increases as K increases, and the information distortion reduces. However, the robustness of the synthesized prior weakens as K increases because the clusters become more and more specific and the amount of data in each cluster tends to decrease. It is desirable to find an optimal K to strike a good balance for this trade-off. To help identifying the optimal K , we introduce the concept of overlapping evidence index (OEI).

Let $\theta |Y_{1},\ldots ,Y_{H}$ denote the random variable corresponding to the synthesized prior $\pi (\theta |Y_{1},\ldots ,Y_{H})$ . The OEI of $\pi (\theta |Y_{1},\ldots ,Y_{H})$ is defined as the weighted sum of overlapping coefficients between the random variable $\theta |Y_{1},\ldots ,Y_{H}$ and each $\theta |Y_{h}$ for h=1,…,H .

(6) $$\mathrm{OEI}(\pi )=\sum_{h=1}^{H}\frac{N_{h}}{N}\cdot OVL(\theta |Y_{1},\ldots ,Y_{H},\theta |Y_{h}),$$

where $N_{h}$ is the sample size of dataset $Y_{h}$ and $N=\sum_{h=1}^{H}N_{h}$ .

OEI(π) lies within the interval [0,1] . It measures the congruence of synthesized prior $\pi (\theta |Y_{1},\ldots ,Y_{H})$ with the information of external data. The higher the OEI(π) , the more congruent information transfers to $\pi (\theta |Y_{1},\ldots ,Y_{H})$ from external data, and the less information distortion occurs. An OEI-based criterion for selecting the optimal number of clusters K , aimed at balancing evidence congruence and robustness, is introduced in Section 4.

## 4. Bayesian clustering prior

### 4.1. The formulation of bayesian clustering prior

For a fixed K , let us assume the optimal partition $S_{K}^{(\mathrm{oci})*}$ is denoted as follows:

$$\begin{array}{rl} & S_{K}^{(\mathrm{oci})*}:\{\theta |Y_{1},\ldots ,\theta |Y_{H}\}\to \{G_{1}^{*},\ldots ,G_{K}^{*}\}=\{\{\theta |Y_{11},\ldots ,\theta |Y_{1n_{1}}\},\ldots ,\{\theta |Y_{K1},\ldots ,\theta |Y_{Kn_{K}}\}\} \end{array}$$

where $n_{m}$ is the number of random variables in cluster $G_{m}^{*}$ , m=1,…,K .

Based on $S_{K}^{(\mathrm{oci})*}$ , the Bayesian clustering prior with K clusters can be constructed as a weighted sum of informative priors synthesized from clusters, $G_{m}^{*}=\{Y_{m1},\ldots ,Y_{mn_{m}}\}$ , m=1,…,K .

(7) $$\pi_{K}(\theta |Y_{1},\ldots ,Y_{H})=\sum_{m=1}^{K}\frac{N_{m}}{N}\cdot \pi (\theta |Y_{m1},\ldots ,Y_{mn_{m}})$$

where $N_{m}$ is the number of observations in cluster $G_{m}^{*}$ , and $N=\sum_{m=1}^{K}N_{m}$ is the size of all external data. Moreover, the prior in equation (7) can be made more robust by adding a weighted weakly informative prior. We refer to this as the robust Bayesian clustering prior:

(8) $$\begin{array}{rl} & \pi_{K}^{R}(\theta |Y_{1},\ldots ,Y_{H})=(1-w)\cdot \sum_{m=1}^{K}\frac{N_{m}}{N}\cdot \pi (\theta |Y_{m1},\ldots ,Y_{mn_{m}})+w\cdot \pi_{0}(\theta ) \end{array}$$

where $\pi_{0}(\theta )$ is a weakly informative prior, w∈[0,1] is the weight of $\pi_{0}(\theta )$ .

In equations (7) and (8), $\pi (\theta |Y_{m1},\ldots ,Y_{mn_{m}})$ can be estimated through various methods, such as traditional Bayesian hierarchical models (BHM), MAP, or power priors. In this article, we choose MAP and robust MAP (rMAP) priors because they can be used in both trial design and data analysis stages. We refer to the resulting priors as Bayesian clustering MAP (BCMAP) and robust Bayesian clustering MAP (rBCMAP), respectively. Weber et al.^11^ developed the R package “RBesT” to implement the sampling of MAP and rMAP through Markov chain Monte Carlo (MCMC). To represent the MAP prior in parametric form, an expectation maximization (EM) algorithm is conducted to approximate the MCMC samples with a parametric mixture distribution. Since BCMAP (rBCMAP) is a weighted sum of MAPs, it can naturally be represented by a parametric mixture distribution as well. Thus, when conjugate MAP priors exist, a mixture of conjugate BCMAP priors can be applied (see the real data example in Section 6.1.1).

The BCMAP prior $\pi_{K}(\theta |Y_{1},\ldots ,Y_{H})$ can be constructed based on $S_{K}^{(\mathrm{oci})*}$ for each K . Then, we can obtain a sequence of $\mathrm{OEI}(\pi_{K})$ , K=1,…,H , which monotonically increase as K increases, that is $0\leq \mathrm{OEI}(\pi_{1})\leq \ldots \leq \mathrm{OEI}(\pi_{H})\leq 1$ . The proof of monotonicity can be found in Theorem 1 in Appendix. We can scale the sequence $\mathrm{OEI}(\pi_{1})\leq \ldots \leq \mathrm{OEI}(\pi_{H})$ by the maximum $\mathrm{OEI}(\pi_{H})$ and refer to $\mathrm{OEI}(\pi_{K})/\mathrm{OEI}(\pi_{H})$ , K=1,…,H , as $\mathrm{SOEI}(\pi_{K})$ . The sequence of $\mathrm{SOEI}(\pi_{K})$ , $\left\{\mathrm{OEI}(\pi_{1})/\mathrm{OEI}(\pi_{H}),\ldots ,\mathrm{OEI}(\pi_{H-1})/\mathrm{OEI}(\pi_{H}),1\right\}$ , denotes the percentage of evidence congruence under each K relative to the extreme case where each distribution is a cluster. A threshold balancing the trade-off of maximizing evidence congruence and minimizing the number of clusters K for robustness can be used to determine the optimal $K^{*}$ . Then, $\pi_{K^{*}}(\theta |Y_{1},\ldots ,Y_{H})$ is the finalized optimal Bayesian clustering prior (see an example in Section 4.3 below). The main advantages of this OEI-based threshold optimizing approach are two-fold: (i) it is straightforward and easy to interpret, as the threshold reflects a balance between the congruence of the synthesized prior with the external data and the robustness to new data, and (ii) a fixed threshold offers a consistent and unified interpretation across various applications. These contrasts with Bayesian nonparametric clustering approaches, where the value of hyperparameters used to determine the number of clusters are often challenging to select and interpret, and may lack consistency across different applications.

### 4.2. Sensitivity analysis and practical guidelines

To provide guidelines for selecting an appropriate threshold, it is essential to understand how the threshold affects clustering performance. We answer this question through a simulation study. The simulation setup consists of two clusters representing low and high parameter values, each containing five subgroups. Specifically, the data are generated as follows: $Y_{li}∼\text{Binomial}(n_{li},\theta_{l})$ and $Y_{hi}∼\text{Binomial}(n_{hi},\theta_{h})$ for i=1,…,5 . We fixed $\theta_{l}=0.2$ and varied $\theta_{h}=0.3,0.4,0.5,0.6$ to create increasing levels of separation between the two cluster, $\theta_{h}-\theta_{l}=0.1,0.2,0.3,0.4$ . To examine the effects of the threshold under different sample sizes, three settings for $n_{li}$ and $n_{hi}$ are considered, where values are drawn from {5,…,20} , {20,…,50} , and {50,…,100} , respectively. We evaluate five threshold values: 0.5,0.6,0.7,0.8,0.9 . For each configuration (i.e., a combination of $\theta_{h}-\theta_{l}$ , sample size, and threshold), we simulate 1000 datasets and compare the average clustering accuracy rate.

The clustering accuracy rate is defined as follows. Suppose there are H objects partitioned into K clusters. For each pair of objects, we assign a label of 1 if they belong to the same cluster and 0 otherwise, resulting in a total of (H2)=H(H−1)/2 labels for any given clustering. We then compare the labels from the estimated clustering (based on a given threshold) to those from the true clustering. Let M denote the number of matching labels between the two. The accuracy rate is then calculated as M/(H2)=2M/(H(H−1)) .

**Figure 2.**
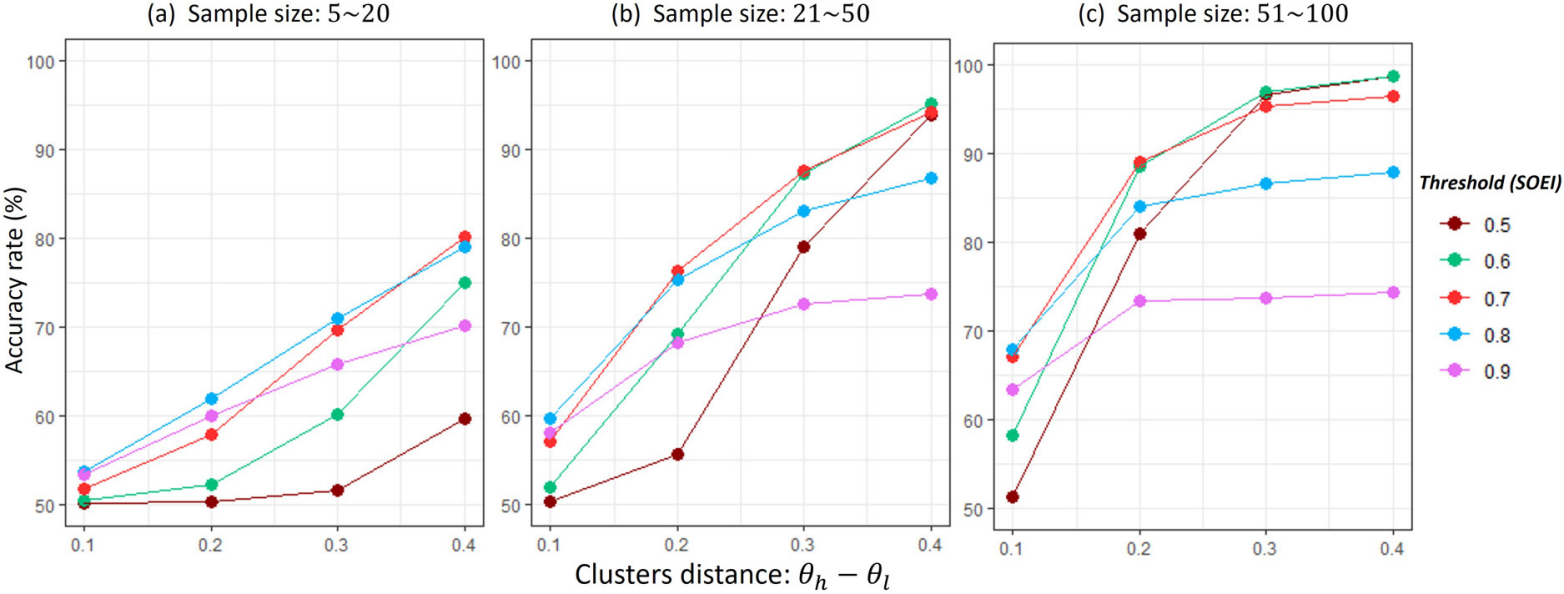
Sensitivity analysis of the scaled OEI (SOEI) threshold.

Several insights emerge from the simulation results in Figure 2. (a) accuracy improves as the sample size increases. (b) Low thresholds (0.5, 0.6) tend to reduce accuracy when clusters are close together and/or the sample size is small. (c) High thresholds (0.9) decrease accuracy when clusters are well separated. Base on these insights, the general guidelines for selecting the threshold are:

- Choose a threshold in the range [0.6,0.7] ;
- Use values closer to 0.7 when the sample size of each study or the average sample size are small ( ≤20 ) or when the gap of clusters is small ( <0.2 );
- Use values closer to 0.6 when the sample size of each study or the average sample size are large ( >20 ) or when the gap of clusters is large ( ≥0.2 ).

### 4.3. An illustrative example

The following example provides an intuitive understanding of the questions: how is BCMAP constructed and how and why does it work? In this example, 12 external datasets were generated by using the model (9). The summary of these datasets is provided in Table 6 in Appendix. It is easy to find the heterogeneity among these datasets that contains two clusters centered at 0.2 and 0.6.

(9) $$\begin{array}{rl} & Y_{1}|\theta ,\sigma^{2},\ldots ,Y_{12}|\theta ,\sigma^{2}∼N(\theta ,\sigma^{2}),\\ & \sigma ∼\mathrm{Halfnormal}(1),\\ & \theta =\phi \cdot (\mu_{c1}+\epsilon_{c1})+(1-\phi )\cdot (\mu_{c2}+\epsilon_{c2}),\\ & \phi ∼\mathrm{Bernoulli}(p=0.5),\;\mu_{c1}=0.2,\;\mu_{c2}=0.6,\\ & \epsilon_{c1}∼N(0,0.05^{2}),\;\epsilon_{c2}∼N(0,0.08^{2}). \end{array}$$

**Figure 3.**
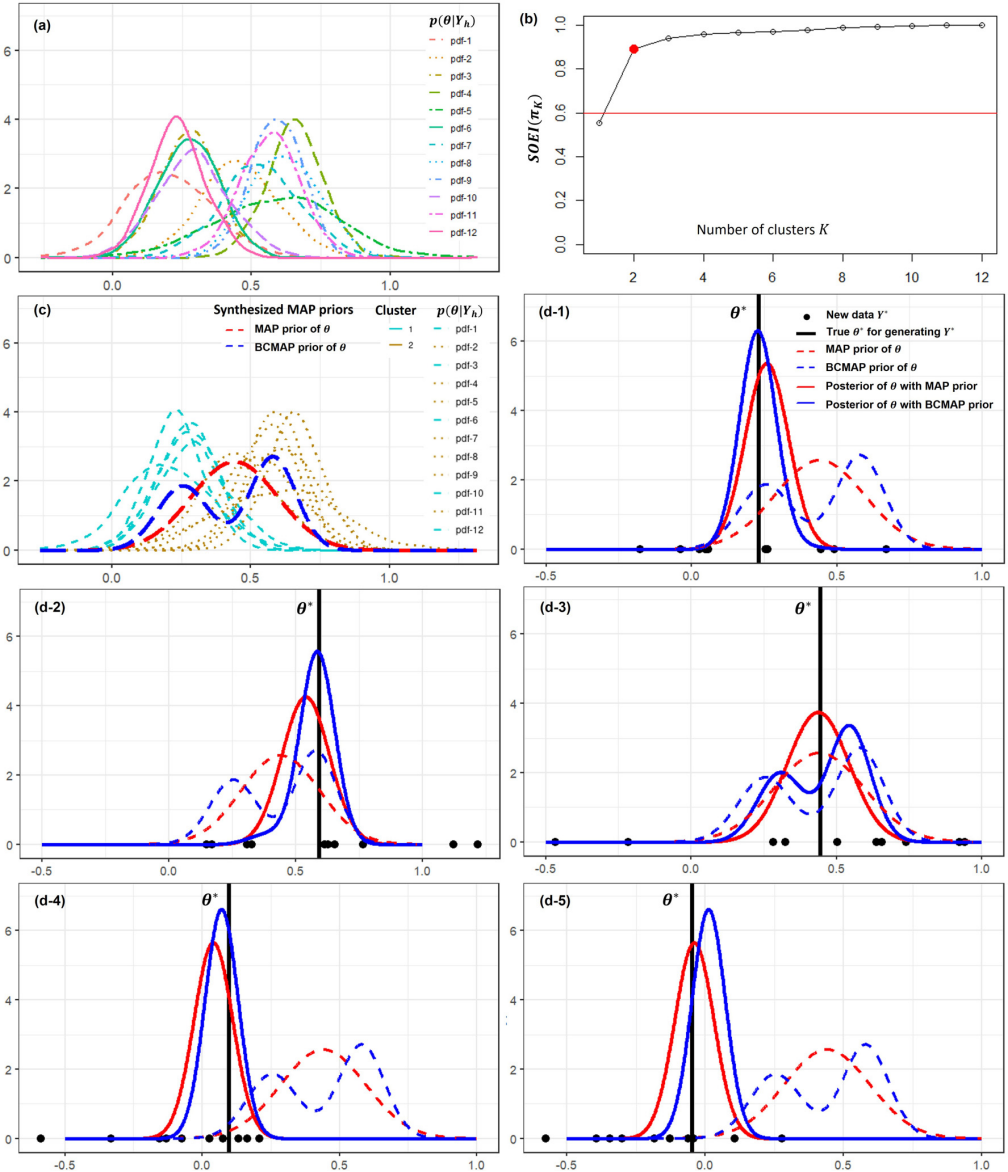
The example of BCMAP and comparisons with MAP. Panel (a) shows the posteriors $p(\theta |Y_{h})\propto L(Y_{h}|\theta )\pi_{0}(\theta )$ , h=1,…,12 , with a weakly informative prior $\pi_{0}(\theta )$ . Panel (b) presents the identification of optimal number of clusters $K^{*}=2$ . Panel (c) exhibits the clustering result and corresponding BCMAP prior, where MAP prior is also provided for comparison. Panels (d-1), (d-2), (d-3), (d-4), and (d-5) show the posteriors, which are obtained by using the new data $Y^{*}$ with various congruence and incongruence to the external data. Panels (d-1) and (d-2) demonstrate the cases that the new data is congruence with the external data. Panels (d-3), (d-4), and (d-5) demonstrate the cases that the new data is incongruence with the external data.

The posteriors $p(\theta |Y_{h})\propto L(Y_{h}|\theta )\pi_{0}(\theta )$ , h=1,…,12 , are shown in Panel (a) of Figure 3. The weakly informative prior $\pi_{0}(\theta )$ is constructed as: $\pi_{0}(\theta )=\int \pi (\theta |\tau )\pi (\tau )\;d\tau$ , where π(θ|τ)∼N(0.4,1/τ) and π(τ)∼gamma(0.01,10) . Given that the average size of the external data is 55, we set the threshold at 0.6 in accordance with the proposed guidelines. Thus, the optimal number of clusters is identified as $K^{*}=2$ as shown in Panel (b). The BCMAP prior is presented in Panel (c), where the corresponding MAP prior is also provided for comparison. The BCMAP prior is congruent with external data, that is RWD. Therefore, if the new data $Y^{*}$ is congruent with the RWD too, which is reasonable, the estimation of $p(\theta |Y^{*})$ will benefit from the information borrowing with the BCMAP prior. To illustrate this, two examples are shown in Panels (d-1) and (d-2) with the new datasets $Y^{*}$ (black dots) generated from $N(\theta^{*},0.3^{2})$ . $\theta^{*}$ (black vertical line) is generated following the θ defined in (9), maintaining congruence between the new data and the external data. We compare the performance of BCMAP and MAP in estimating $\theta^{*}$ . In Panel (d-1), $\theta^{*}$ is near the center at 0.2. The posterior with BCMAP prior outperforms MAP prior in the estimations of both location (bias) and scale (variance). In Panel (d-2), $\theta^{*}$ is near the center at 0.6. BCMAP prior again outperforms MAP priors in both location and scale estimation. However, when the new data is incongruent with the external data in a certain way, BCMAP may perform worse than MAP, as illustrated in Panel (d-3). Notably, this does not imply that BCMAP consistently underperforms in all incongruent scenarios. For example, in Panel (d-4), BCMAP still outperforms MAP despite the presence of incongruence. In Panel (d-5), although MAP provides a more accurate estimate of the location, BCMAP exhibits a smaller variance.

The example highlights the strengths and limitations of the proposed Bayesian clustering prior. Specifically, the superiority of this approach depends on two key conditions, which are commonly met in practice.

- **Heterogeneity among external datasets:** The Bayesian clustering MAP (BCMAP) prior is particularly advantageous when there is heterogeneity among the external datasets. In the absence of heterogeneity, BCMAP typically identifies a single cluster, causing the BCMAP prior to degenerate to the MAP prior (see Figure 8 in Appendix).
- **Congruence between new data and external data:** For the BCMAP to perform well, the new data $Y^{*}$ must be congruent with the external data. Specifically, the data generating process of $Y^{*}$ should align with that of the external datasets, even if it is not identical. For example, $Y^{*}$ may follow a distribution that corresponds to one of the components in the mixture distribution of the external data. If this condition is not met, the BCMAP may not work well. In fact, none of the priors will work well if there are severe prior-data conflicts. In this case, the rBCMAP can provide a partial remedy by incorporating robustness into the prior construction (see Section 5.1).

## 5. Simulation studies

In this section, we conduct comprehensive simulation studies to demonstrate the advantages of BCMAP (rBCMAP) in both parameter estimation and hypothesis testing compared to commonly used priors, such as MAP, rMAP, NPP (Normalized Power Prior), and MEM. Note: for both rBCMAP and rMAP, the weight for robustness (weight of weakly informative component) is set to 0.5.

### 5.1. Parameter estimation

The simulation study for parameter estimation follows the prior comparison framework described in Section 4.3. We examine external data under three different heterogeneity scenarios: one, two, and three clusters. For each scenario, we conduct 1000 simulation runs. In each run, we generate new data samples and compute the posterior distributions using various priors.

**Figure 4.**
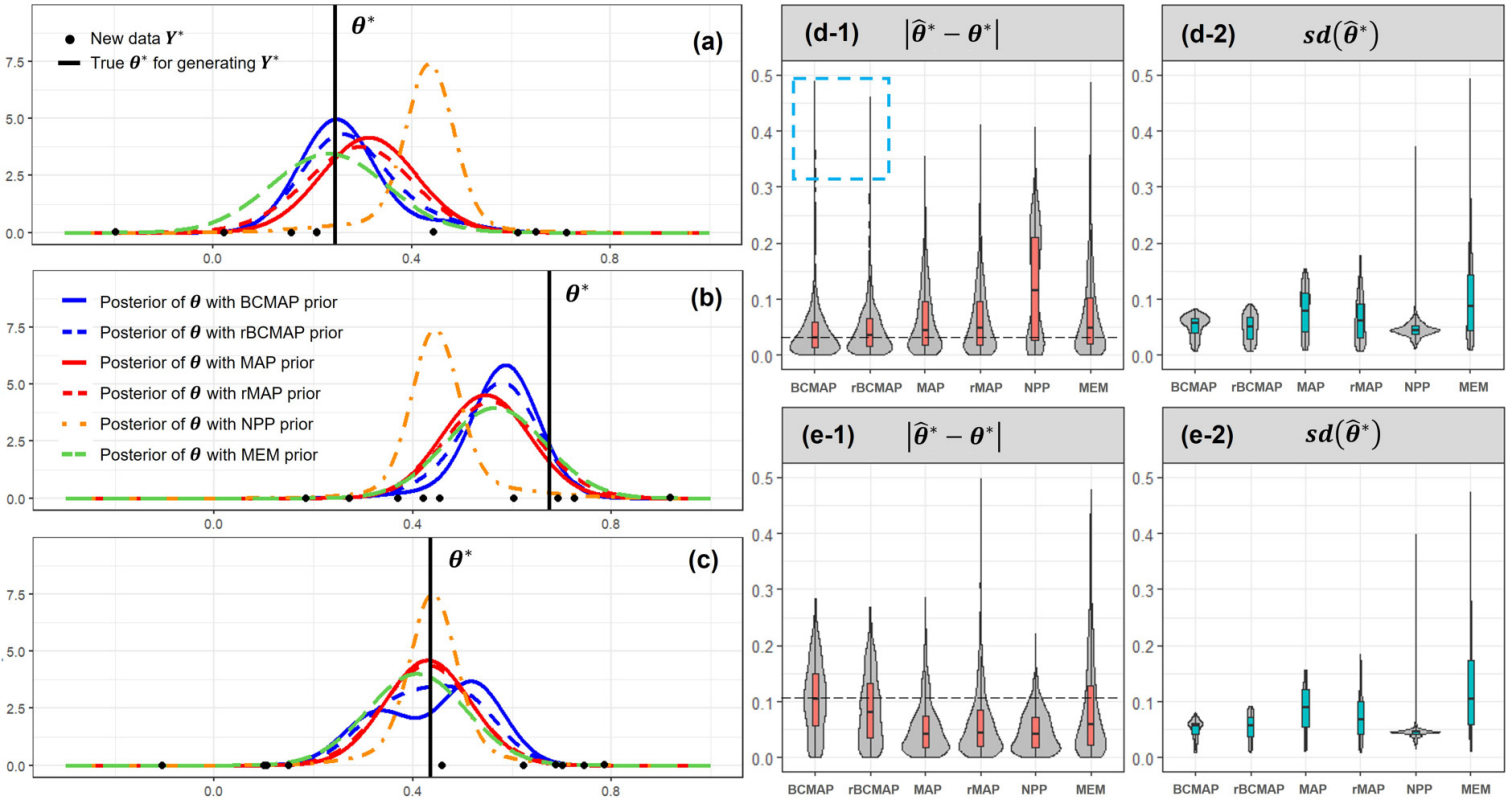
Estimation of new $\theta^{*}$ from posteriors computed with different priors. In Panels (a) and (b), $\theta^{*}$ is congruent with the external data, located near the cluster centers at 0.2 and 0.6, respectively. In contrast, Panel (c) illustrates an incongruent scenario, where $\theta^{*}$ lies between the two cluster centers. Panels (d-1) and (d-2) present the bias and standard deviation of the estimator ${\hat{\theta }}^{*}$ under the congruent cases. Panels (e-1) and (e-2) show the corresponding results for the incongruent case, where $\theta^{*}$ is positioned between the two centers. All results in (d-1) through (e-2) are based on 1000 simulations, each with 10 observations of $Y^{*}$ .

In the two-cluster scenario, we use the 12 external datasets described in Section 4.3, whose posteriors are displayed in Panel (a) of Figure 3. The new data consists of 10 observations generated from a normal distribution $N(\theta^{*},0.3^{2})$ , where $\theta^{*}$ is drawn from the model (9). Then, the posteriors are then calculated based on $Y^{*}$ using different priors. Intuitive comparison can be conducted by considering the posterior estimation of the location (bias) and scale (standard deviation) of estimate ${\hat{\theta }}^{*}$ . Let us examine three examples shown in Panels (a), (b), and (c) of Figure 4. In Panels (a) and (b), $\theta^{*}$ is close to the centers 0.2 or 0.6, indicating the congruence of the new data $Y^{*}$ with the external data. BCMAP and rBCMAP outperform other methods with less bias and lower variance of the estimate ${\hat{\theta }}^{*}$ . However, Panel (c) exhibits the opposite scenario where $\theta^{*}$ is located in the middle of the two centers, indicating the incongruence between the new data $Y^{*}$ and the external data. This results in a worse performance of BCMAP compared to the other methods. But, such scenarios as in Panel (c) are rare under the assumption that the new data $Y^{*}$ is congruent with the external data, i.e., RWD. A valuable observation in Panel (c) is that rBCMAP can enhance the robustness of BCMAP when the assumption of congruence is violated. The results from 1000 simulation runs under both congruent and incongruent scenarios are presented in Panels (d-1) through (e-2). Panels (d-1) and (d-2) correspond to the congruent scenario. Panel (d-1) shows the empirical distribution of the absolute bias, $|{\hat{\theta }}^{*}-\theta^{*}|$ , where ${\hat{\theta }}^{*}$ is the mode of the posterior. In this setting, BCMAP and rBCMAP exhibit lower bias compared to the other methods. Panel (d-2) displays the empirical distribution of the standard deviation, $sd({\hat{\theta }}^{*})$ . Excluding NPP, BCMAP and rBCMAP perform better than all other methods. Although NPP has the lowest standard deviation, it has the highest bias in Panel (d-1). In the incongruent scenarios shown in Panels (e-1) and (e-2), BCMAP exhibits the highest bias among all methods. However, with a robustness weight of 0.5, rBCMAP effectively reduces this bias, consistent with the observation in Panel (c).

The summary of the estimation results for both the congruence and incongruence scenarios is presented in Table 1. Regarding the root mean square error (RMSE) criterion, both BCMAP and rBCMAP shows a smaller RMSE (0.077 and 0.076) compared to the MAP and rMAP (0.088 and 0.092) in the congruence scenario, aligning with the results shown in Panels (d-1) and (d-2) of Figure 4. Sometimes, the influence of outliers, as highlighted in Panel (d-1) of Figure 4 (indicated by the blue dashed rectangle), may cause inflation of the RMSE ( see Tables 7 and 8 in Appendix). In these case, we can remove the top 5% or 10% of data (outliers) to reduce the inflation. Regarding the coverage rate of the 95% highest (posterior) density interval (HDI), in the congruence scenario, both BCMAP and rBCMAP outperform all other methods. In the incongruence scenario, however, the HDI coverage of BCMAP experiences a significant drop, falling below that of other methods. Nevertheless, the rBCMAP provides a substantial remedy for this issue.

**Table 1.** Comparison of RMSE and the HDI coverage rate under the scenarios of congruence and incongruence shown in panel (d-1), (d-2) and (e-1), (e-2) in figure 4. “remove top 5%” means that calculates RMSE after removing the top 5% of data (outliers). “95% HDI CR” is the 95% highest (posterior) density interval (HDI) coverage rate about the true parameter.

Scenarios	Criteria	Data	BCMAP	MAP	rBCMAP	rMAP	NPP	MEM
Congruence	RMSE	All	0.077	0.088	0.076	0.092	0.156	0.105
		Remove top 5%	0.047	0.073	0.052	0.073	0.145	0.082
	95% HDI CR	All	0.972	0.955	0.972	0.963	0.807	0.953
Incongruence	RMSE	All	0.124	0.071	0.107	0.089	0.063	0.136
		Remove top 5%	0.115	0.059	0.098	0.067	0.053	0.104
	95% HDI CR	All	0.906	0.953	0.938	0.946	0.995	0.928

The simulation presented in Figure 4 focuses solely on the two-cluster heterogeneity scenario of the external datasets and keeps the new data with 10 observations. To enable a more comprehensive comparison, we extend the study in two directions: (1) in addition to the two-cluster scenario, we examine cases with one and three clusters; (2) we evaluate the effect of varying the size of $Y^{*}$ , considering sizes of 5, 10, 15, 20, 25, and 30. In the one-cluster scenario, 10 external datasets are generated from the model (10), representing homogeneity among the datasets.

(10) $$\begin{array}{rl} & Y_{1}|\theta ,\sigma^{2},\ldots ,Y_{10}|\theta ,\sigma^{2}∼N(\theta ,\sigma^{2}),\\ & \sigma ∼Halfnormal(1),\\ & \theta =\mu +\epsilon ,\;\mu =0.6,\;\epsilon ∼N(0,0.06^{2}). \end{array}$$

While the three-clusters scenario includes 25 external datasets generated from the model (11), reflecting different heterogeneity from the two-clusters scenario.

(11) $$\begin{array}{rl} & Y_{1}|\theta ,\sigma^{2},\ldots ,Y_{25}|\theta ,\sigma^{2}∼N(\theta ,\sigma^{2}),\\ & \sigma ∼Halfnormal(1),\\ & \theta =I(c=c_{1})\cdot (\mu_{c1}+\epsilon_{c1})+I(c=c_{2})\cdot (\mu_{c2}+\epsilon_{c2})+I(c=c_{3})\cdot (\mu_{c3}+\epsilon_{c3}),\\ & c∼multinomial(p_{c1}=0.3,p_{c2}=0.4,p_{c3}=0.3),\\ & \mu_{c1}=0.2,\;\mu_{c2}=0.6,\;\mu_{c3}=1,\\ & \epsilon_{c1}∼N(0,0.05^{2}),\;\epsilon_{c2}∼N(0,0.06^{2}),\\ & \epsilon_{c3}∼N(0,0.08^{2}). \end{array}$$

A summary of the three scenarios, including the number of clusters, number of datasets, and sample sizes within each dataset, is provided in Table 9 in the Appendix. The posteriors (with a weakly informative prior), clustering results, and synthesized BCMAP and rBCMAP priors for the one-cluster and three-cluster scenarios are presented in Figures 8 and 9, respectively, in the Appendix.

**Figure 5.**
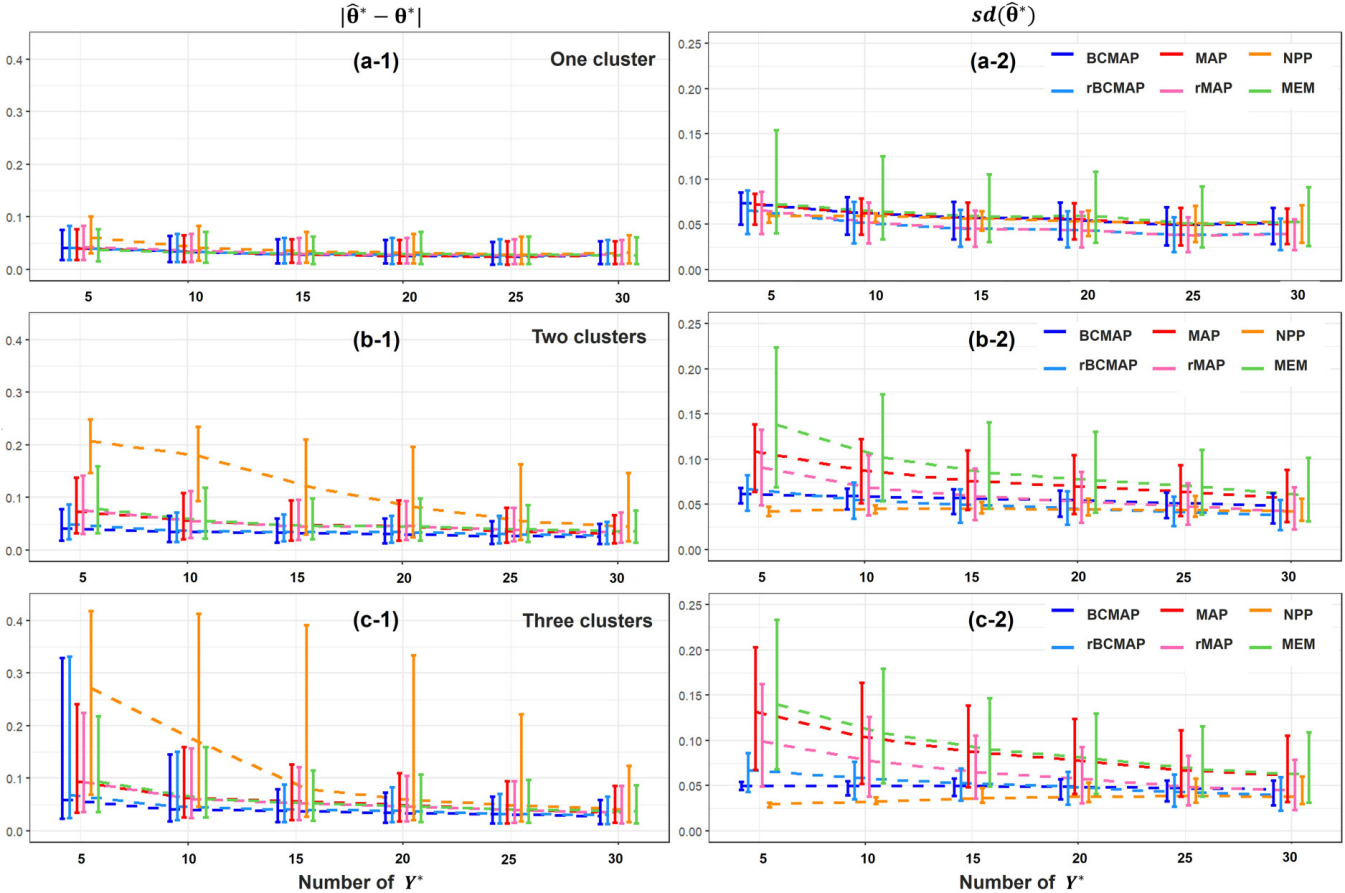
Comparison of parameter estimation under different heterogeneous scenarios with various size of new observations $Y^{*}$ . Panels in left and right columns present the bias and standard deviation of estimate ${\hat{\theta }}^{*}$ , respectively. Panels in top row show the result under homogeneous scenario. Panels in middle row present the result under two clusters scenario, where the true new $\theta^{*}$ is near the cluster centers 0.2 or 0.6. Panels in bottom row exhibit the result under three clusters scenario, where the true new $\theta^{*}$ is near the cluster centers 0.2, 0.6, or 1.

The simulation results are presented in Figure 5. The Panels in the left and right columns illustrate the comparison of bias $|{\hat{\theta }}^{*}-\theta^{*}|$ and variance $sd({\hat{\theta }}^{*})$ , respectively. In the one-cluster scenario, the MAP and rMAP priors are identical to the BCMAP and rBCMAP priors, respectively, as shown in Panels (a-1) and (a-2). In the two- and three-clusters scenarios, when the new data $Y^{*}$ is congruent with the external data, the BCMAP and rBCMAP priors outperform other methods. Notably, the NPP exhibits poor performance, characterized by the smallest $sd({\hat{\theta }}^{*})$ but the largest bias. An interesting observation is that as the size of $Y^{*}$ increases, all methods tend to converge to similar results because the new data begins to dominate the prior. The summary (RMSE and 95% HDI coverage rate) for the two clusters and three clusters scenarios (excluding the one cluster scenario, as BCMAP and rBCMAP are equivalent to MAP and rMAP in this case) is presented in Table 7 and Table 8, respectively.

In summary, if the new data $Y^{*}$ is congruent with external data that exhibits heterogeneity, BCMAP and rBCMAP priors provide more accurate parameter estimation, with lower bias and variance, than commonly used methods. When the new data $Y^{*}$ is incongruent with the external data, both BCMAP and rBCMAP may perform worse than alternative methods. But, rBCMAP can provide a partial remedy, as demonstrated in Panel (e-1) of Figure 4 and Tables 1, 7, and 8.

### 5.2. Hypothesis testing

In this section, we compare the performance of different priors in hypothesis testing. All priors are constructed from the external datasets used in the illustrative example (see Panel (a) of Figure 3) in Section 4.3. We design a prospective trial with two groups: control and treatment. Observations are generated from $N(\theta_{c},\sigma^{2})$ and $N(\theta_{t},\sigma^{2})$ , where σ=0.3 and $\theta_{t}=0.4$ . Since the possible values of $\theta_{c}$ are centered at 0.2 and 0.6 by the model (9), we consider two scenarios: $\theta_{c1}=0.2$ and $\theta_{c2}=0.6$ . Correspondingly, we are interested in two hypothesis tests:

1.
$H_{0}:\theta_{t}\leq \theta_{c1},\;H_{1}:\theta_{t}>\theta_{c1}$

2.
$H_{0}:\theta_{t}\geq \theta_{c2},\;H_{1}:\theta_{t}<\theta_{c2}$ .

In the simulation, we investigate both frequentist and Bayesian methods. The frequentist method is a one-sided two-sample t-test. We considered two control vs. treatment recruitment ratios, 10:30 and 30:30 . For Bayesian methods, to study the effect of information borrowing from synthesized priors, we use the same data with control vs. treatment ratio 10:30 and evaluate the gain in power by incorporating external control data. Corresponding to the two hypothesis tests, the decision rule (reject $H_{0}$ ) and operational characteristics in Bayesian methods are defined as follows:

1. Decision rule:
$Pr(\theta_{t}>\theta_{c1}|Y_{t}^{*},Y_{c1}^{*},Y_{1},\ldots ,Y_{12})>\eta$ ;
  Type 1 error rate:
$Pr(Pr(\theta_{t}>\theta_{c1}|Y_{t}^{*},Y_{c1}^{*},Y_{1},\ldots ,Y_{12})>\eta |H_{0})$ ;
  Power:
$Pr(Pr(\theta_{t}>\theta_{c1}|Y_{t}^{*},Y_{c1}^{*},Y_{1},\ldots ,Y_{12})>\eta |H_{1})$ .
2. Decision rule:
$Pr(\theta_{t}<\theta_{c2}|Y_{t}^{*},Y_{c2}^{*},Y_{1},\ldots ,Y_{12})>\eta$ ;
  Type 1 error rate:
$Pr(Pr(\theta_{t}<\theta_{c2}|Y_{t}^{*},Y_{c2}^{*},Y_{1},\ldots ,Y_{12})>\eta |H_{0})$ ;
  Power:
$Pr(Pr(\theta_{t}<\theta_{c2}|Y_{t}^{*},Y_{c2}^{*},Y_{1},\ldots ,Y_{12})>\eta |H_{1})$ ,

where η∈[0.5,1) is an adjustable threshold used to control the type 1 error rate under 5%. In the calculation of $Pr(\theta_{t}>\theta_{c1}|Y_{t}^{*},Y_{c1}^{*},Y_{1},\ldots ,Y_{12})$ and $Pr(\theta_{t}<\theta_{c2}|Y_{t}^{*},Y_{c2}^{*},Y_{1},\ldots ,Y_{12})$ , we need to find the joint posterior $p(\theta_{t},\theta_{c}|Y_{t}^{*},Y_{c}^{*},Y_{1},\ldots ,Y_{12})$ , where $\theta_{c}=\{\theta_{c1},\theta_{c2}\}$ and $Y_{c}^{*}=\{Y_{c1}^{*},Y_{c2}^{*}\}$ . Since $\theta_{t}$ and $\theta_{c}$ are independent, the joint posterior can be expressed as follows:

(12) $$\begin{array}{rl} & p(\theta_{t},\theta_{c}|Y_{t}^{*},Y_{c}^{*},Y_{1},\ldots ,Y_{12})\propto L(Y_{t}^{*}|\theta_{t})\pi_{0}(\theta_{t})L(Y_{c}^{*}|\theta_{c})\pi (\theta_{c}|Y_{1},\ldots ,Y_{12}) \end{array}$$

where $\pi_{0}(\theta_{t})$ is a weakly informative prior indicating trivial prior knowledge about the treatment group, and $\pi (\theta_{c}|Y_{1},\ldots ,Y_{12})$ denotes the synthesized priors from the 12 external datasets.

The simulation results are shown in Table 2. For the frequentist method, the results of both hypothesis tests (1) and (2) show a dramatic reduction in power as the size of the control group decreases from 30 to 10. (Note: the frequentist method does not involve clustering or information borrowing.) Regarding Bayesian methods, except for the case of NPP in hypothesis test (1), information borrowing from synthesized priors can improve the test power compared to the frequentist 10:30 trial. However, the simulation results also illustrate that the quality of the prior plays a critical role. NPP performs best in test (2) but worst in test (1). MEM and rMAP perform similarly, offering only modest gains in power. MAP provides a greater improvement but remains less effective than rBCMAP and BCMAP. Overall, BCMAP achieves the highest performance, increasing power by more than 35%, and is comparable to the frequentist 30:30 trial. In sum, Bayesian cluster priors enable the incorporation of accurate information from heterogeneous external data. When the new data $Y_{c}^{*}$ is generally congruent with the external data, borrowing information from Bayesian cluster priors can effectively improve the operational characteristics of hypothesis testing.

**Table 2.** Comparison of operation characteristic in hypothesis testing. Two hypothesis tests: (1) $H_{0}:\theta_{t}\leq \theta_{c1},\;H_{1}:\theta_{t}>\theta_{c1}$ and (2) $H_{0}:\theta_{t}\geq \theta_{c2},\;H_{1}:\theta_{t}<\theta_{c2}$ . For the Bayesian methods, the control vs. treatment ratio is 10:30 , but incorporating external control data in the construction of the prior distribution.

	$\theta_{c1}=0.2$		$\theta_{c1}=0.6$	
Methods	Type 1 Error	Power	Type 1 Error	Power
*Control : Treatment = 30 : 30*				
Frequentist	0.044	0.810	0.055	0.817
*Control : Treatment = 10 : 30*				
Frequentist	0.055	0.567	0.053	0.550
Bayesian				
MEM	0.055	0.610	0.054	0.580
NPP	0.004	0.439	0.008	0.835
MAP	0.049	0.663	0.051	0.693
rMAP	0.053	0.568	0.051	0.608
BCMAP	**0.050**	**0.783**	**0.051**	**0.773**
rBCMAP	0.048	0.668	0.051	0.727

**Table 3.** Studies on acupoint P6 stimulation for preventing nausea. The variables are: **id:** trial id number. **study (author):** first author of the study. **year:** study year. **ai:** number of patients experiencing nausea in treatment (wrist acupuncture point P6 treatment) group. **n1i:** total number of patients in treatment group. **in cluster:** partition $p(\theta |Y_{h})$ into the corresponding cluster.

id	study (author)	year	ai ( $r_{h}$ )	n1i ( $N_{h}$ )	in cluster
*External/historical data*
1	Dundee	*1986*	**3**	**25**	2
2	Gieron	*1993*	**11**	**30**	3
3	Allen	*1994*	**9**	**23**	3
4	Andrzejowski	*1996*	**11**	**18**	3
5	Ferrera-Love	*1996*	**1**	**30**	1
6	Ho	*1996*	**1**	**30**	1
7	Duggal	*1998*	**69**	**122**	3
8	Alkaissi	*1999*	**9**	**20**	3
9	Harmon	*1999*	**7**	**44**	2
10	Agarwal	*2000*	**18**	**100**	2
11	Harmon	*2000*	**4**	**47**	1
12	Zarate	*2001*	**28**	**110**	2
*Current data*
13	Agarwal	*2002*	**5**	**50**	–
14	Alkaissi	*2002*	**32**	**135**	–
15	Rusy	*2002*	**24**	**40**	–
16	Wang	*2002*	**16**	**50**	–

## 6. Real data examples

### 6.1. Acupuncture trials

Postoperative nausea and vomiting are common complications following surgery and anesthesia. As an alternative to drug therapy, acupuncture has been studied as a potential treatment in several trials.^20^ The dataset “dat.lee2004” in the R package “metadat”^21^ contains the results from 16 clinical trials examining the effectiveness of wrist acupuncture point P6 treatment for preventing postoperative nausea. Patient level (covariate) information is not available. A detailed description of the dataset is provided in Table 3. The columns “ai” and “n1i” correspond to $r_{h}$ and $N_{h}$ , respectively, where $N_{h}$ denotes the number of patients in study h , and $r_{h}$ indicates the number of patients who experienced postoperative nausea. The data are modeled using a Binomial distribution: $Binomial(N_{h},\theta )$ , where θ represents the probability of a patient experiencing nausea. We denote the observed data for study h as $Y_{h}=\{r_{h},N_{h}\}$ . There are four studies (id = 13, 14, 15, and 16) were conducted in 2002. To illustrate the application of the proposed prior in data analysis, we treat each of these four datasets as the current data, and the other 12 studies conducted before 2002 as external (historical) data.

**Figure 6.**
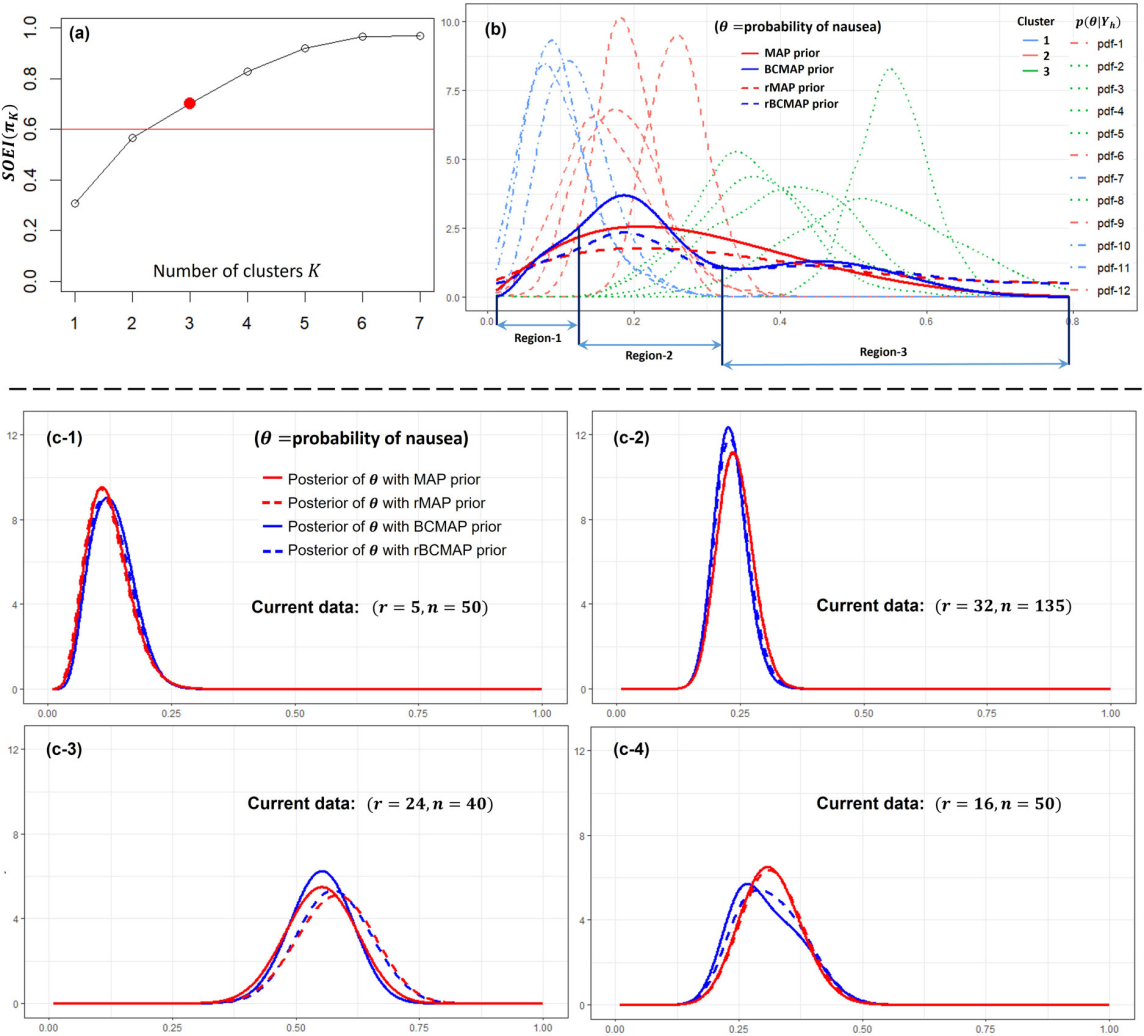
Studies on the effectiveness of wrist acupuncture point P6 treatment for preventing postoperative nausea. Panel (a): the optimal number of cluster K=3 . Panel (b) the corresponding BCMAP, rBCMAP, MAP, and rMAP. Panels (c-1) to (c-4): the posteriors with different priors and current data.

#### 6.1.1. Prior for study design

In trial design study, our goal is to construct an informative prior $\pi (\theta |Y_{1},\ldots ,Y_{12})$ from the external data to provide useful prior information for the new trial. To achieve this goal, we begin by examining the posteriors of the external data using a vague conjugate prior, π(θ)=Beta(0.5,0.5) . The posterior distributions are straightforward to derive: $\theta |Y_{h}∼Beta(0.5+y_{h},0.5+N_{h}-y_{h}),\;h=1,\ldots ,12$ , as shown in Panel (b) of Figure 6. The results reveal substantial heterogeneity across the studies, making this dataset well-suited for applying the Bayesian clustering prior. Next, we determine the optimal number of clusters. Given that the average sample size is approximately 50, we set the threshold at 0.6 in accordance with the guidelines outlined in Section 4.2. The optimal number of clusters is identified as 3 as shown in Panel (a). The corresponding BCMAP and rBCMAP priors are presented in Panel (b), alongside the MAP and rMAP priors for comparison. Their parameterized forms are listed below:

(13) $$\begin{array}{rl} & \pi_{MAP}(\theta |Y_{1},\ldots ,Y_{12})=Beta(2.2,5.5) \end{array}$$

(14) $$\begin{array}{rl} & \pi_{rMAP}(\theta |Y_{1},\ldots ,Y_{12})=0.5\cdot Beta(2.2,5.5)+0.5\cdot Beta(1,1) \end{array}$$

(15) $$\begin{array}{rl} & \pi_{BCMAP}(\theta |Y_{1},\ldots ,Y_{12})=0.18\cdot Beta(4.7,38.1)+0.47\cdot Beta(10.6,40.6)+0.35\cdot Beta(9.7,11.5). \end{array}$$

(16) $$\begin{array}{rl} & \pi_{rBCMAP}(\theta |Y_{1},\ldots ,Y_{12})=0.09\cdot Beta(4.7,38.1)+0.23\cdot Beta(10.6,40.6)+0.18\cdot Beta(9.7,11.5)+0.5\cdot Beta(1,1). \end{array}$$

Based on equations (13) and (15), compared to MAP prior, the BCMAP prior has the advantage that its effective sample size (ESS) can be estimated in a finer way. From equation (13), we know that the ESS of $\pi_{MAP}(\theta |Y_{1},\ldots ,Y_{12})$ over the entire region [0,1] is 2.2+5.5≈8 . Let us check the ESS of $\pi_{BCMAP}(\theta |Y_{1},\ldots ,Y_{12})$ from equation (15) and Panel (b): in region 1, $ESS_{1}\approx 0.18\cdot (4.7+38.1)\approx 8$ ; in region 2, $ESS_{2}\approx 0.47\cdot (10.6+40.6)\approx 24$ ; in region 3, $ESS_{3}\approx 0.35\cdot (9.7+11.5)\approx 7$ . This offers valuable prior information for the design of future trials. For example, acupuncture treatment appears more likely to reduce the probability of postoperative nausea to below 30%, with a concentration in the range of 15% to 30%. For the rMAP and rBCMAP priors, the effect of robustification results in an ESS of 0.5⋅(1+1)=1 .

#### 6.1.2. Prior for data analysis

Applying the constructed priors to the current datasets (id = 13, 14, 15, and 16), we can obtain the posteriors of θ as shown in Panels (c-1) to (c-4). Overall, the resulting posteriors are not substantially affected by the choice of prior, likely because the relatively large sample sizes in the current datasets dominate the posterior inference. However, in Panel (c-4), the BCMAP prior noticeably shifts the posterior toward the middle cluster (around 0.2), while the rBCMAP prior slightly counteracts this effect, drawing the posterior closer to those obtained under the MAP and rMAP priors.

### 6.2. Potassium supplementation to reduce diastolic blood pressure

In this section, we consider continuous endpoint data with the normal distribution. We use the dataset “dat.curtin2002” from the R package “metadat”, which includes 21 cross-over studies evaluating the effect of potassium supplementation on reducing diastolic blood pressure. This dataset does not contain patient-level information. We perform a preliminary data cleaning step by removing two outlier studies with mean values of −13.1 and −8.0 . The resulting cleaned dataset is provided in Table 4.

**Table 4.** Studies on potassium supplementation to reduce diastolic blood pressure. The variables are: **id:** trial id number. **study (author):** first author of the study. **year:** study year. **N:** number of patients in each study. **mean:** the mean of each study. **SE:** the standard error of each study. **in cluster:** partition $p(\theta |Y_{h})$ into the corresponding cluster.

id	study (author)	year	N	mean	SE	in cluster
*External/historical data*
1	Skrabal	*1981*	20	**–4**.**5**	**2.1**	2
2	Skrabal	*1981*	20	**–0**.**5**	**1.7**	4
3	MacGregor	*1982*	23	**–4**.**0**	**1.9**	2
4	Khaw	*1982*	20	**–2**.**4**	**1.1**	3
5	Richards	*1984*	12	**–1**.**0**	**3.4**	4
6	Smith	*1985*	20	**0**.**0**	**1.9**	4
7	Kaplan	*1985*	16	**–5**.**8**	**1.6**	1
8	Zoccali	*1985*	23	**–3**.**0**	**3.0**	3
9	Matlou	*1986*	36	**–3**.**0**	**1.5**	3
10	Barden	*1986*	44	**–1**.**5**	**1.4**	4
11	Poulter a	*1986*	19	**2**.**0**	**2.2**	5
12	Grobbee	*1987*	40	**–0**.**3**	**1.5**	4
13	Mullen	*1990*	24	**3**.**0**	**2.0**	6
14	Mullen	*1990*	24	**1**.**4**	**2.0**	5
15	Valdes	*1991*	24	**–3**.**0**	**2.0**	3
16	Barden	*1991*	39	**–0**.**6**	**0.6**	4
17	Overlack	*1991*	12	**3**.**0**	**2.0**	6
*Current data*
18	Smith	*1992*	22	**–1**.**7**	**2.5**	–
19	Fotherby	*1992*	18	**–6**.**0**	**2.5**	–

**Figure 7.**
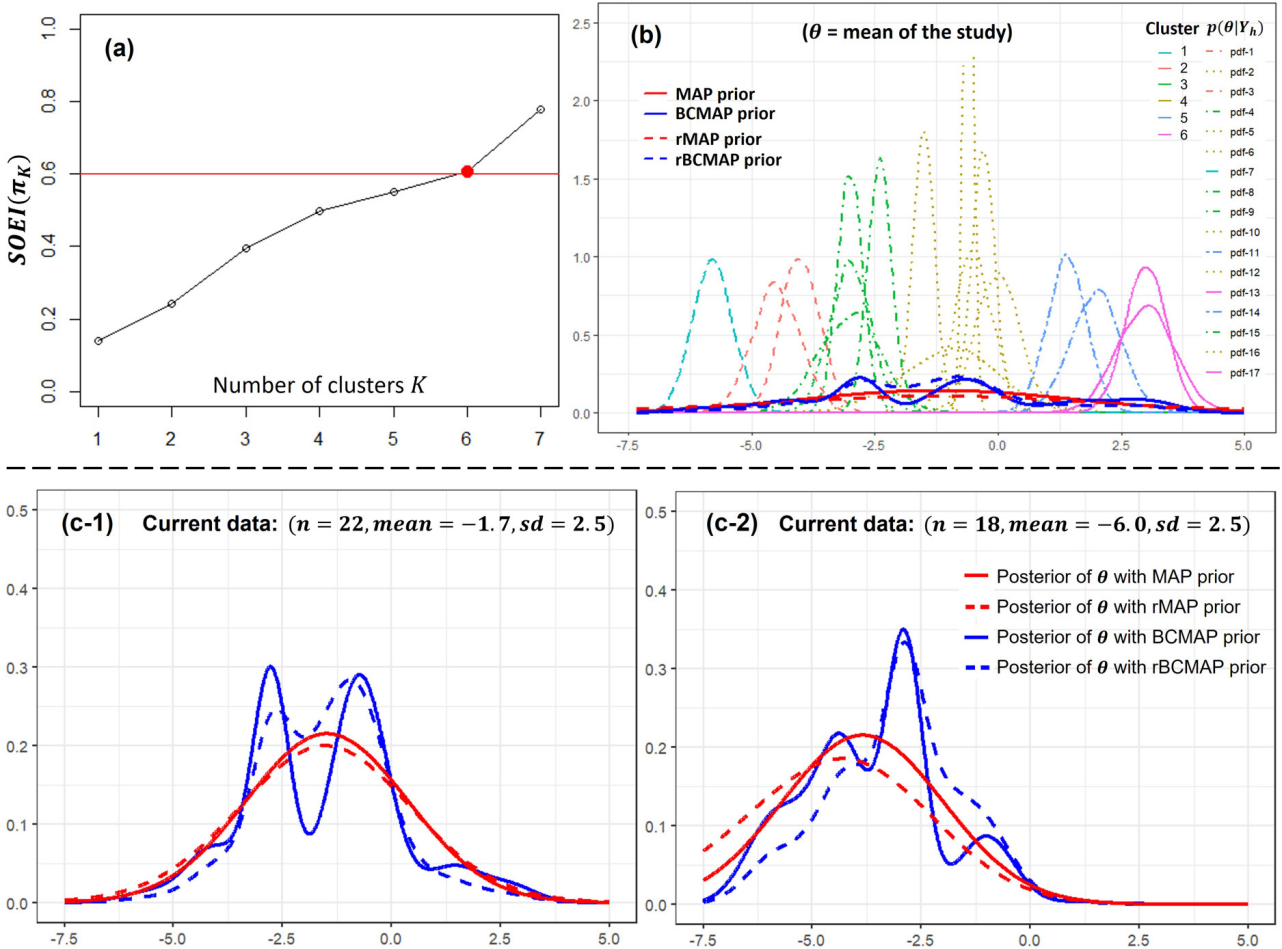
Studies on potassium supplementation to reduce diastolic blood pressure. Panel (a): the optimal number of cluster K=6 . Panel (b) the corresponding BCMAP, rBCMAP, MAP, and rMAP. Panels (c-1) and (c-2): the posteriors with different priors and current data.

**Figure 8.**
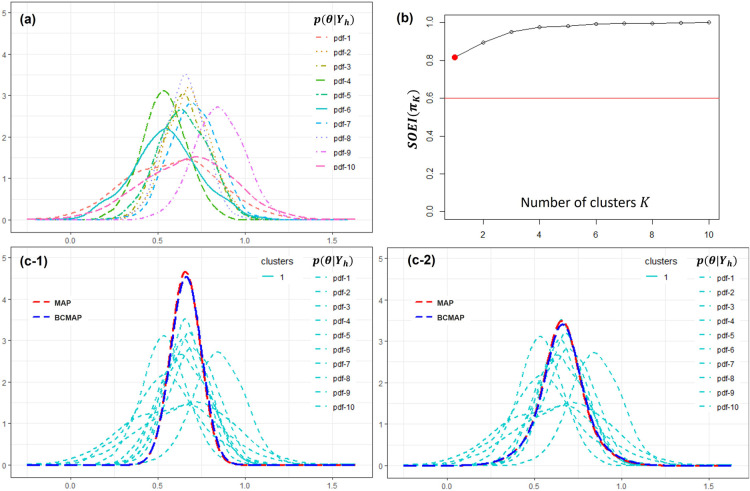
External data with one cluster. Panel (a): the posteriors with weakly informative prior. Panel (b): the optimal number of clusters. Panel (c-1): the synthesized MAP and BCMAP. Panel (c-2): the synthesized rMAP and rBCMAP.

**Figure 9.**
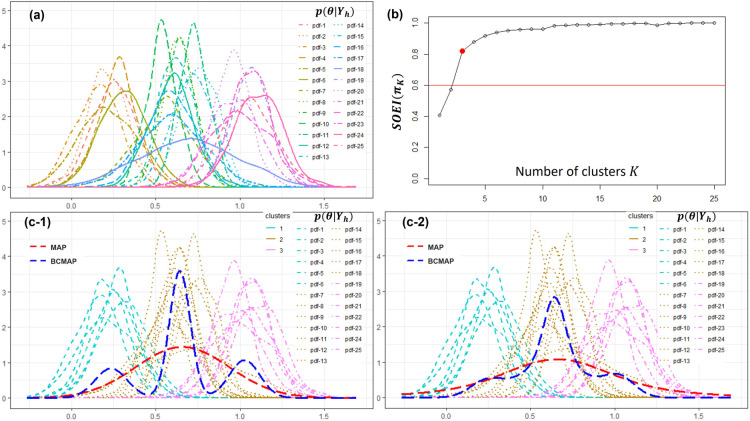
External data with three clusters. Panel (a): the posteriors with weakly informative prior. Panel (b): the optimal number of clusters. Panel (c-1): the synthesized MAP and BCMAP. Panel (c-2): the synthesized rMAP and rBCMAP.

Similar to the acupuncture example, in order to simulate data analysis, we pick out the two studies from 1992 (id = 18, 19) as the current data and those conducted before 1992 as external data (17 external datasets). Given a prior $N(\theta_{0},\sigma_{0}^{2})$ , the corresponding posteriors can be computed as follows:

$$\theta |{\bar{y}}_{h},s_{h}^{2}∼N\left(\frac{\sigma_{0}^{2}}{\frac{s_{h}^{2}}{n_{h}}+\sigma_{0}^{2}}{\bar{y}}_{h}+\frac{s_{h}^{2}/n_{h}}{\frac{s_{h}^{2}}{n_{h}}+\sigma_{0}^{2}}\theta_{0},{\left(\frac{1}{\sigma_{0}^{2}}+\frac{n_{h}}{s_{h}^{2}}\right)}^{-1}\right)$$

where, $n_{h}$ , ${\bar{y}}_{h}$ , and $s_{h}^{2}$ denote the sample size, sample mean, and sample variance for dataset h , respectively. We set the prior mean and variance as $\theta_{0}=\frac{1}{H}\sum_{h=1}^{H}{\bar{y}}_{h}$ and $\sigma_{0}=10\cdot {\left(\sum_{h=1}^{H}s_{h}^{2}\right)}^{-2}$ , in order to specify a weakly informative prior $N(\theta_{0},\sigma_{0}^{2})$ . The resulting posterior distributions are shown in Panel (b) of Figure 7.

Since the posterior distributions are well spread, indicating substantial separation between potential clusters, we select a threshold of 0.6 in accordance with the guidelines in Section 4.2. The optimal number of clusters is identified as K=6 as shown in Panel (a) of Figure 7. The corresponding BCMAP and rBCMAP priors are shown in Panel (b), alongside MAP and rMAP for comparison. Compared to MAP and rMAP, the BCMAP and rBCMAP priors exhibit more pronounced concentration around the cluster centers, particularly near -0.7 and -2.8. This prior information suggests that future studies are more likely to observe the values of pressure reduction centered around these two values.

The posterior distributions of θ , based on the constructed priors and current data, are displayed in Panels (c-1) and (c-2). Unlike the acupuncture example, the current data in this case are relatively small, which leads the prior to exert a stronger influence on the posterior. The rBCMAP serves to moderate the impact of the BCMAP, yielding a smoother posterior distribution closer to MAP and rMAP.

## 7. Discussion

Clustering plays a crucial role in synthesizing informative priors from heterogeneous multisource external data. Leveraging the concept of the overlapping coefficient, we introduce the OCI, OEI, K-Means algorithm, and an OEI-based criterion to identify the optimal clustering. Based on it, a Bayesian clustering prior can be constructed and applied during both the trial design and data analysis stages. Simulation studies validate its advantages.

Effectively borrowing information from external (historical) data remains an active area of research. The proposed Bayesian clustering prior represents an effort to address the challenges of information borrowing from heterogeneous multisource external data. Several potential research directions of this approach merit further investigation:

First, this study focuses on the case where θ is one-dimensional. Since the definition of the overlapping coefficient (OVL) extends naturally to higher dimensions, We believe the proposed method can also be extended to multi-dimensional θ . However, the computational aspects of OVL in higher dimensions, which impact the calculation of OCI and OEI, require more careful consideration.

Second, we do not incorporate covariate information in the current study. However, in the era of precision medicine, covariates are becoming increasingly important. A straightforward way to integrate covariates into the Bayesian clustering prior is through their inclusion in posterior estimation, for instance via the MAP approach. Further research is needed to explore more advanced strategies for incorporating covariates, such as regression-based adjustments or propensity score matching.

Third, this paper adopts a fixed weight of w=0.5 , representing an equal balance between the informative prior derived from external data and a weakly informative component. While convenient, this choice may not be optimal across all scenarios. Data-driven methods for estimating the weight dynamically could offer greater flexibility and potentially enhance performance.

Finally, beyond the K-Means-based approach used here, alternative clustering techniques, such as Gaussian mixture models, could be employed to simultaneously perform clustering and prior construction. These alternatives deserve further exploration and comparative evaluation.

## Appendix

**Table 5.** Description of symbols and abbreviations.

Symbol / Abbreviation	Description
$Y_{h}$ , h=1,…,H	External datasets.
$p(\theta |Y_{h})$	Posterior distribution of θ given external data $Y_{h}$
$\theta |Y_{h}$	The random variable corresponding to the posterior $p(\theta |Y_{h})$ .
$p(\theta |Y_{1},\ldots ,Y_{H})$	Constructed prior distribution of θ given external data $Y_{1},\ldots ,Y_{H}$ .
$\theta |Y_{1},\ldots ,Y_{H}$	The random variable corresponding to the posterior $p(\theta |Y_{1},\ldots ,Y_{H})$ .
OVL(X,Y)	Overlapping coefficient between random variables X and Y .
${\mathrm{OCI}}_{K}$	Overlapping clustering index of a K partition.
$S_{K}$	K -partition of $\theta |Y_{h}$ , h=1,…,H .
$G_{m}$	The cluster m in the partition $S_{K}$ .
$\Theta_{m}$	The centroid random variable of cluster $G_{m}$ .
$S_{K}^{(\mathrm{oci})*}$	The optimal partition identified by maximizing $OCK_{K}$ .
$S_{K}^{(\mathrm{km})*}$	The optimal partition identified by K-Means clustering algorithm.
OEI(π)	Overlapping evidence index of the prior π(θ) .
$\pi_{K}(\theta |Y_{1},\ldots ,Y_{H})$	The Bayesian clustering prior with K clusters given the external data.
$\pi_{K}^{R}(\theta |Y_{1},\ldots ,Y_{H})$	The robust Bayesian clustering prior with K clusters given the external data.
$\mathrm{SOEI}(\pi_{K})$	The sequence of scaled overlapping evidence index.
MAP	Meta-analytic predictive prior.
rMAP	Robust meta-analytic predictive prior.
BCMAP	Bayesian clustering meta-analytic predictive prior.
rBCMAP	Robust Bayesian clustering meta-analytic predictive prior.
NPP	Normalized Power Prior.
MEM	Multisource exchangeability model prior.
$\theta^{*}$ , $Y^{*}$	The value of parameter and corresponding random sample in the future (new) study.

Theorem 1 Monotonicity of OEIs
For the BCMAP priors $\pi_{K}$ , K=1,…,H , the sequence $\{OEI(\pi_{K}){\}}_{K=1}^{H}$ increases monotonically with the increase of K , i.e., $0\leq OEI(\pi_{1})\leq \ldots \leq OEI(\pi_{H})\leq 1$ .
Proof. Without losing generality, let us compare $OEI(\pi_{K})$ and $OEI(\pi_{K+1})$ , K<H . Correspondingly, we need to consider the difference between $S_{K}^{(\mathrm{oci})*}$ and $S_{K+1}^{(\mathrm{oci})*}$ . For an arbitrary cluster in $S_{K}^{(\mathrm{oci})*}$ , say $G_{m}^{(K)*}$ , we split it to two clusters say $G_{m}^{\left(K+1\right)}$ and $G_{K+1}^{\left(K+1\right)}$ , where $G_{m}^{(K)*}=G_{m}^{\left(K+1\right)}\cup G_{K+1}^{\left(K+1\right)}$ . The new partition is denoted as $S_{K+1}^{\left(\mathrm{oci}\right)}$ . In each cluster, we assume that the random variables $\theta |Y_{h}$ are homogeneous and use them to construct a MAP prior. For $G_{m}^{(K)*}$ , the corresponding MAP random variable is denoted as $\theta |G_{m}^{(K)*}$ . For $G_{m}^{\left(K+1\right)}$ and $G_{K+1}^{\left(K+1\right)}$ , they are presented as $\theta |G_{m}^{\left(K+1\right)}$ and $\theta |G_{K+1}^{\left(K+1\right)}$ . Since the homogeneity of cluster $G_{m}^{\left(K+1\right)}$ and cluster $G_{K+1}^{\left(K+1\right)}$ are greater than that of cluster $G_{m}^{(K)*}$ , it follows that:

$$\begin{array}{rl} \sum_{h=1}^{n_{m}}\frac{N_{mh}}{N}\cdot OVL\left(\theta |G_{m}^{(K)*},\theta |Y_{mh}\right) & \leq \sum_{h=1}^{n_{m}}\frac{N_{mh}}{N}\cdot OVL\left(\theta |G_{m}^{\left(K+1\right)},\theta |Y_{mh}\right)\cdot I\left(Y_{mh}\in G_{m}^{\left(K+1\right)}\right)\\ & \;+\sum_{h=1}^{n_{m}}\frac{N_{mh}}{N}\cdot OVL\left(\theta |G_{K+1}^{\left(K+1\right)},\theta |Y_{mh}\right)\cdot I\left(Y_{mh}\in G_{K+1}^{\left(K+1\right)}\right). \end{array}$$

By equation (6) in the definition of OEI, we know that

$$\mathrm{OEI}(\pi_{K})\leq \mathrm{OEI}\left(\pi_{S_{K+1}^{\left(\mathrm{oci}\right)}}\right).$$

By definition of the Overlapping Clustering Index (OCI), $S_{K+1}^{(\mathrm{oci})*}$ denotes the partition that maximizes the overall within-cluster homogeneity among all K+1 partitions (including $S_{K+1}^{\left(\mathrm{oci}\right)}$ ). This implies:

$$\mathrm{OEI}\left(\pi_{S_{K+1}^{\left(\mathrm{oci}\right)}}\right)\leq \mathrm{OEI}\left(\pi_{S_{K+1}^{(\mathrm{oci})*}}\right)=\mathrm{OEI}(\pi_{K+1}).$$

Therefore, $\mathrm{OEI}(\pi_{K})\leq \mathrm{OEI}(\pi_{K+1})$ holds. Note: This proof is applied to the rBCMAP as well.

**Table 6.** Summary of 12 external datasets grouped into two clusters: a low cluster centered at 0.2 and a high cluster centered at 0.6.

Dataset ( $Y_{h}$ )	Cluster	Cluster center ( $\mu_{c}$ )	Size ( $n_{h}$ )	Mean ( $\theta_{h}$ )	Standard deviation ( $\sigma_{h}$ )
$Y_{1}$	Low	0.2	32	0.220	0.560
$Y_{2}$	High	0.6	36	0.443	0.230
$Y_{3}$	Low	0.2	69	0.206	1.559
$Y_{4}$	High	0.6	62	0.656	0.071
$Y_{5}$	High	0.6	16	0.562	0.129
$Y_{6}$	Low	0.2	62	0.172	1.715
$Y_{7}$	High	0.6	36	0.515	0.461
$Y_{8}$	High	0.6	47	0.583	1.265
$Y_{9}$	High	0.6	98	0.518	0.687
$Y_{10}$	Low	0.2	43	0.289	0.446
$Y_{11}$	High	0.6	78	0.542	1.224
$Y_{12}$	Low	0.2	81	0.225	0.360

**Table 7.** Comparison of RMSE and the HDI coverage rate under two clusters scenario with different number of $Y^{*}$ . “remove top 5%” means that calculates RMSE after removing the top 5% of data (outliers). “95% HDI CR” is the 95% highest (posterior) density interval (HDI) coverage rate of the true parameter.

Size of $Y^{*}$	Criteria	Data	BCMAP	MAP	rBCMAP	rMAP	NPP	MEM
5	RMSE	All	0.100	0.115	0.101	0.129	0.214	0.155
			Remove top 5%	0.070	0.101	0.075	0.106	0.202	0.122
	95% HDI CR	All	0.950	0.934	0.943	0.923	0.327	0.905
10	RMSE	All	0.089	0.098	0.086	0.102	0.192	0.120
			Remove top 5%	0.055	0.084	0.059	0.085	0.179	0.093
	95% HDI CR	All	0.968	0.949	0.964	0.950	0.717	0.939
15	RMSE	All	0.080	0.085	0.076	0.090	0.158	0.100
			Remove top 5%	0.051	0.072	0.054	0.073	0.147	0.078
	95% HDI CR	All	0.963	0.947	0.961	0.949	0.805	0.940
20	RMSE	All	0.078	0.085	0.076	0.090	0.149	0.104
			Remove top 5%	0.049	0.071	0.054	0.071	0.133	0.080
	95% HDI CR	All	0.966	0.950	0.962	0.948	0.846	0.940
25	RMSE	All	0.066	0.075	0.067	0.080	0.127	0.089
			Remove top 5%	0.044	0.062	0.049	0.064	0.111	0.067
	95% HDI CR	All	0.964	0.949	0.963	0.950	0.877	0.945
30	RMSE	All	0.058	0.070	0.059	0.073	0.120	0.080
			Remove top 5%	0.040	0.056	0.043	0.057	0.105	0.060
	95% HDI CR	All	0.962	0.945	0.958	0.943	0.893	0.943

**Table 8.** Comparison of RMSE and the HDI coverage rate under three clusters scenario with different number of $Y^{*}$ . “remove top 5%” means that calculates RMSE after removing the top 5% of data (outliers). “95% HDI CR” is the 95% highest (posterior) density interval (HDI) coverage rate of the true parameter.

Size of $Y^{*}$	Criteria	Data	BCMAP	MAP	rBCMAP	rMAP	NPP	MEM
5	RMSE	All	0.234	0.211	0.235	0.216	0.316	0.231
			Remove 10% outliers	0.183	0.153	0.185	0.150	0.276	0.147
	95% HDI CR	All	0.881	0.860	0.850	0.879	0.384	0.879
10	RMSE	All	0.192	0.168	0.190	0.169	0.294	0.165
			Remove 10% outliers	0.135	0.109	0.135	0.105	0.257	0.106
	95% HDI CR	All	0.928	0.906	0.899	0.917	0.565	0.929
15	RMSE	All	0.145	0.132	0.148	0.129	0.263	0.132
			Remove 10% outliers	0.075	0.086	0.081	0.082	0.221	0.083
	95% HDI CR	All	0.941	0.924	0.927	0.936	0.687	0.938
20	RMSE	All	0.132	0.113	0.131	0.111	0.237	0.111
			Remove 10% outliers	0.061	0.074	0.064	0.071	0.187	0.071
	95% HDI CR	All	0.936	0.926	0.934	0.934	0.767	0.933
25	RMSE	All	0.127	0.112	0.126	0.110	0.211	0.108
			Remove 10% outliers	0.050	0.067	0.054	0.067	0.156	0.064
	95% HDI CR	All	0.940	0.923	0.924	0.929	0.810	0.937
30	RMSE	All	0.111	0.099	0.110	0.099	0.191	0.099
			Remove 10% outliers	0.041	0.058	0.045	0.057	0.131	0.059
	95% HDI CR	All	0.943	0.931	0.942	0.940	0.836	0.941

**Table 9.** The simulated external datasets. Homogeneous scenario: one cluster, heterogeneous scenarios: two clusters and three clusters.

Cluster	Number of datasets	Sample size in each dataset
1	10	17, 71, 62, 72, 57, 31, 71, 94, 57, 15
2	12	32, 36, 69, 62, 16, 62, 36, 47, 98, 43, 78, 81
3	25	37, 57, 42, 54, 30, 40, 26, 82, 96, 92, 99, 57,
		73, 69, 60, 43, 19, 10, 52, 68, 35, 24, 67, 38, 33
